# Genomic analysis of *Colletotrichum camelliae* responsible for tea brown blight disease

**DOI:** 10.1186/s12864-023-09598-6

**Published:** 2023-09-06

**Authors:** Linghong Kong, Jiao Chen, Kaili Dong, Karim Shafik, Wenxing Xu

**Affiliations:** 1 National Key Laboratory for Germplasm Innovation & Utilization of Horticultural Crops, Huazhong Agricultural University, Wuhan, China; 2 Hubei Hongshan Laboratory, Huazhong Agricultural University, Wuhan, 430070 Hubei China; 3 College of Plant Science and Technology, Huazhong Agricultural University, Wuhan, 430070 Hubei China; 4 Key Lab of Plant Pathology of Hubei Province, Wuhan, 430070 Hubei China; 5 Department of plant pathology, Faculty of Agriculture, Alexandria University, Alexandria, 21526 Egypt

**Keywords:** *Colletotrichum*, *Colletotrichum camelliae*, Genome, Virulence factors, Pathogenicity, Tea brown blight disease

## Abstract

**Background:**

*Colletotrichum camelliae*, one of the most important phytopathogenic fungi infecting tea plants (*Camellia sinensis*), causes brown blight disease resulting in significant economic losses in yield of some sensitive cultivated tea varieties. To better understand its phytopathogenic mechanism, the genetic information is worth being resolved.

**Results:**

Here, a high-quality genomic sequence of *C. camelliae* (strain LT-3-1) was sequenced using PacBio RSII sequencing platform, one of the most advanced Three-generation sequencing platforms and assembled. The result showed that the fungal genomic sequence is 67.74 Mb in size (with the N50 contig 5.6 Mb in size) containing 14,849 putative genes, of which about 95.27% were annotated. The data revealed a large class of genomic clusters potentially related to fungal pathogenicity. Based on the Pathogen Host Interactions database, a total of 1698 genes (11.44% of the total ones) were annotated, containing 541 genes related to plant cell wall hydrolases which is remarkably higher than those of most species of *Colletotrichum* and others considered to be hemibiotrophic and necrotrophic fungi. It’s likely that the increase in cell wall-degrading enzymes reflects a crucial adaptive characteristic for infecting tea plants.

**Conclusion:**

Considering that *C. camelliae* has a specific host range and unique morphological and biological traits that distinguish it from other species of the genus *Colletotrichum*, characterization of the fungal genome will improve our understanding of the fungus and its phytopathogenic mechanism as well.

**Supplementary Information:**

The online version contains supplementary material available at 10.1186/s12864-023-09598-6.

## Background

The genus *Colletotrichum* (Family Glomerellaceae, Ascomycota) has significant and varied groups, such as saprophytes and endophytes in addition to plant pathogens that infect a range of plant hosts [1–4]. However, some *Colletotrichum* species (e.g., *C. fioriniae* and *C. gloeosporioides*) can infect insects in hemlock and citrus plants [5]. In addition, several species (e.g., *C. dematium*) could cause some human diseases including keratitis and subcutaneous infections [6, 7]. More than 100 Colletotrichum species have been identified as phytopathogenic species that cause rot, foliar blight, and anthracnose diseases in a number of commercial crops, causing major yield losses every year across the globe [1, 8–10]. Therefore, the *Colletotrichum* genus has been evaluated as the eighth-most significant group of plant pathogenic fungus worldwide, based on perceived scientific and economic importance[2].

Tea plant (*Camellia sinensis* [L.] O. Kuntze), which originated in southwestern China, is considered to be one of the most economically important crops worldwide. Due to tea products, it is ranked as the second most popular beverage in the world after water and one of the three main non-alcoholic beverages, along with coffee and cocoa. There are numerous diseases that have an impact on tea production. However, *C*. *camelliae* Massea (sexual taxon: *Glomerella cingulata* ‘f. sp. camelliae’ Dickens & R.T.A. Cook)causes tea brown blight which is one of the most prevalent and significant diseases affecting tea plants [11–13]. Tea brown blight disease is characterized with cloudy symptoms composed by large gray-white lesions on tea leaves, and the infected plants frequently exhibit dieback of the tender branches, defoliation, and the entire plants of some sensitive varieties may even die. Moreover, many other *Colletotrichum* spp. (e.g., *C. camelliae*, *C. gloeosporioides*, *C. alienum*, *C. boninense*, *C. cliviae*, *C. fioriniae*, *C. fructicola*, *C. karstii*, *C. siamense*, *C. henanense*, and *C. jiangxiense*) have been isolated from *Camellia* plants. Among them, some *Colletotrichum* species (e.g., *C. siamense*, *C. henanense*, and *C. fructicola*) can induce anthracnose disease on tea plants [14–18]. Since *C. camelliae* differs from other *Colletotrichum* species and is related to tea plants with high economic importance, it is crucial to resolve its genetic information.

In the present study, a high-quality genomic sequence of *C. camelliae* was sequenced, assembled, and annotated. The genomic information potentially related to the fungal pathogenicity was investigated. In addition, both synteny and phylogenetic relationships between *C. camelliae* and other pathogens were analyzed. Phylogenetically, the result showed that *C. camelliae* is closely related to *C. gloeosporioides*, whereas the data revealed a large class of diverse genes or other functional clusters, resulting in separating *C. camelliae*, as a specific species infecting tea plants, from the remaining species of the genus *Colletotrichum*.

## Results

### Genome sequencing and assembly

**Fig. 1:**
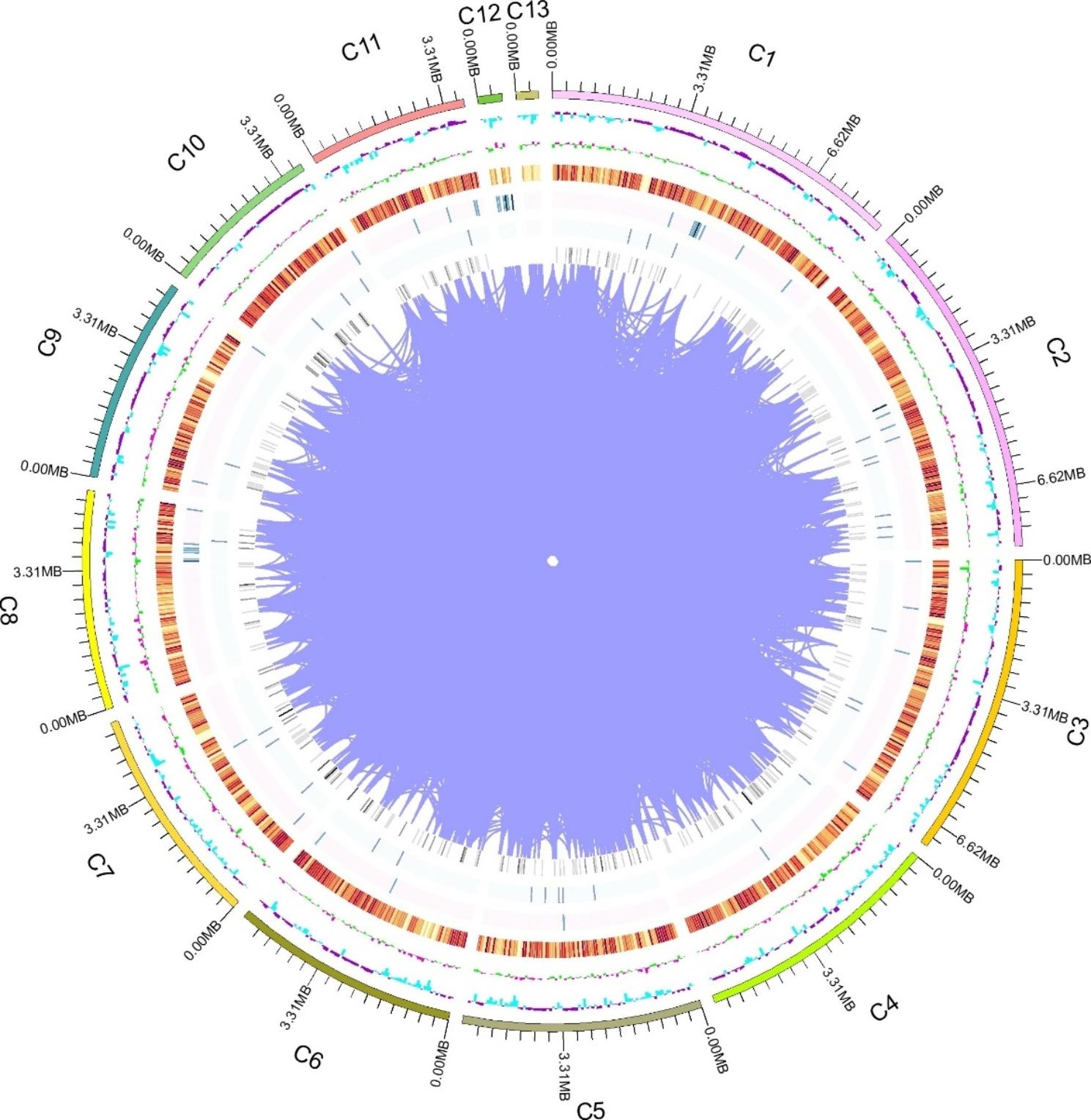
The circular genome diagram of *C. camelliae* strain LT-3-1. The outer loop indicates the position coordinates of the genome sequence. From outside to inside: the position coordinates of the genome sequence (C1 to C13, arc-shaped bars), GC content (teeth bars), protein-encoding genes (colored lines), rRNA (grey lines), snRNA (grey lines), and tRNA (grey lines). All these were calculated with a window of 20,000 bp and a step length of 20,000 bp. For GC content, the inward blue part indicates that the GC content of this region is lower than the average GC content of the whole genome, the outward purple part is the opposite, the inward green part indicates that the content of G in this area is lower than that of C, and the outward pink part is the opposite; for the gene density, rRNA, snRNA, and tRNA, the colors refer to their density with darker color indicating a higher density

The nucleic acids of of *C. camelliae* strain LT-3-1, stored in our lab that was isolated from tea plants showing typical brown blight symptoms (Figure S1) [38], were extracted and then subjected to whole genome sequencing using the PacBio sequencing platform. After removing both repeats and low-quality reads, a total of 490,505 reads with 4.65 Gb (a mean read size of 9479 bp and an N50 size of 13,044 bp) were obtained and assembled into 21 contigs (67.74 Mb in length with a maximum of 8.54 Mb in size and a N50 in size of 5.69 Mb; with GC content of 48.98%) (Table 1; Fig. 1).

**Table 1:** Assembly and gene prediction statistics for the genome of *Colletotrichum camelliae* strain LT-3-1.

Category	Number
Contigs	21
Maximum length (bp)	8539808
N50 length (bp)	5691745
Total length (bp)	67743009
GC content (%)	48.98
Gene number	14849
Total gene length (bp)	23042074
Gene average length (bp)	1552
Total gene size/Genomic size (%)	34.01

### Gene prediction

By predicting genes, ncRNAs, and repeats of the genome, a total of 14,849 genes with 23.04 Mb in size (about 34.01% of the whole genome) were detected in the coding regions, and the average length of the genes is 1.552 kb (Table 1). The results revealed that the numbers of dispersed repeats sequences (DRs) include 3269 long terminal repeat elements (LTRs), 1229 DNA transposons, 923 long interspersed nuclear elements (LINEs), 42 short interspersed nuclear elements (SINEs), 39 rolling circles (RC), 27 regions with unknown functions, and 5529 interspersed repeated sequences (Table 2). According to the results obtained from the tandem repeat sequences (TRs), the genome of *C. camelliae* showed a total of 11,131 satellite DNAs were found, including 9339 minisatellite DNAs with 10–60 bp repeats, and 1972 microsatellite DNAs with repeats of 2–6 bp (Table 2). Noticeably, a total of 12,705 TRs with a total size of 615,135 bp (0.908% of the whole genome) were detected. Moreover, a total of 404 tRNAs in addition to 98 rRNAs, including 65 5 S-rRNAs, 1 18 S-rRNAs, 2 sRNAs, and 30 snRNAs, were detected. However, no 5.8 S rRNAs, 28 S-rRNAs, or miRNAs were detected (Table S1).

**Table 2:** Statistical results for dispersed repeats sequences and tandem repeat sequences in the genome of *C. camelliae* LT-3-1.

Type	Number	Total length (bp)	Proportion in genome (%)	Average length (bp)/ Repeat size (bp)
LTR	3269	1575008	2.3250	493
DNA	1229	279932	0.4132	236
LINE	923	151314	0.2234	171
SINE	42	2894	0.0043	69
RC	39	2638	0.0039	69
Unknown	27	1995	0.0029	74
TR	12705	615135	0.9080	1-877
Minisatellite DNA	9339	423722	0.6255	10–60
Microsatellite DNA	1792	80761	0.1192	2–6

### Gene annotation

**Fig. 2:**
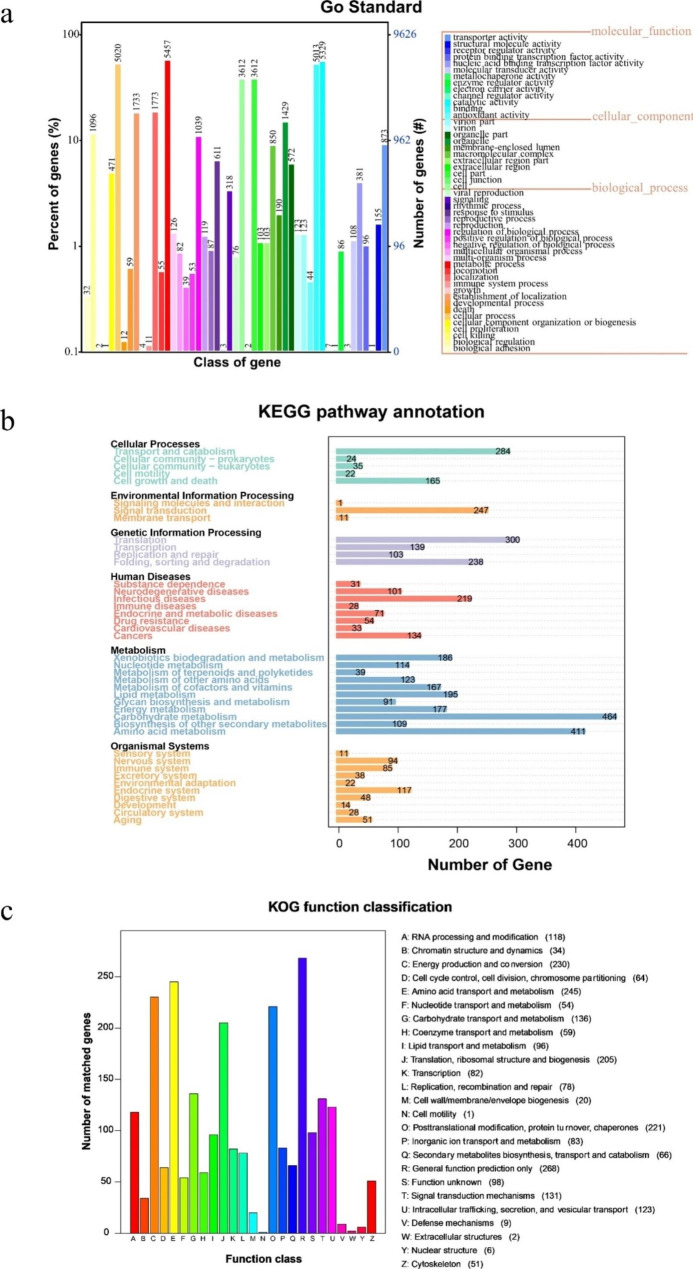
Gene annotation of *C. camelliae* genome. **(a)** Gene Ontology (GO) functional annotation. Abscissas and ordinate axis refer to the classification and percentage of genes, respectively. **(b)** the Kyoto Encyclopedia of Genes and Genomes (KEGG) function annotation. Abscissas and ordinate axis refer to gene number and the related pathways, respectively. **(c)** Clusters of orthologous groups of proteins (KOG) functional classification in *C. camelliae*. Abscissas and ordinate axis refer to functional classification and gene number, respectively

The assembled contigs were further subjected to gene annotation based on GO, KEGG, KOG, NR, TCDB, Pfam, SwissProt, CAZy, CYP450, PHI, and DFVF databases (Table S2). A total of 14,849 protein-coding genes were predicted, among them, 14,147 (95.27%) genes could be annotated. In addition, a total of 9625 (64.82%) of the total predicted genes were annotated in the GO database (Fig. 2a).

The protein coding sequences of the *C. camelliae* genome were divided into three major classes and 49 subclasses, including coding regions that are involved in biological processes (18,279 coding regions with 25 subclasses), cellular components (10,719 coding regions with 11 subclasses), and molecular functions (12,097 coding regions with 13 subclasses). Among those involved in biological processes, a total of 5457 coding genes were found to be related to metabolic processes, while a total of 5020 genes were involved in cellular processes. However, for those related to cellular components, a total of 471 coding genes (the greatest number) were involved in cells and cell parts; a total of 5329 and 5013 coding genes involved in molecular functions were found to have catalytic and binding activities, respectively.

Based on the results of the KEGG database, a total of 12,181 genes of *C. camelliae* were annotated, with 7029 genes enriched in 363 metabolic pathways (Fig. 2b) including the genes (with different abundances) related to cellular processes, environmental information processing, genetic information processing, human diseases, metabolism, or organic systems. Those involved in metabolism had the largest (2076) number of genes, of which, a total of 464 genes were related to carbohydrate metabolism.

According to the results of the KOG database, a total of 2248 genes of *C. camelliae* were annotated, which were found to be mainly concentrated in general function prediction (268 genes), amino acid transport and metabolism (245 genes), energy production and conversion (230 genes), post-translation modification, protein turnover, and chaperones (221 genes), translation, ribosomal structure and biogenesis (205 genes), and others (Fig. 2c).

It revealed a total of 14,147, 9625, and 3795 genes in the NR, Pfam, and Swiss-Prot databases, accounting for 95.27%, 64.82%, and 25.56% of total *C. camelliae* genes, respectively. In the NR database, *C. camelliae* genes matched those of *C. gloeosporioides* (12,556 genes), *C. orbiculare* (198 genes), and *C. incanum* (179 genes), ranking at the top three fungal species.

### Genomic clusters related to the fungal pathogenicity

#### Transporter

**Fig. 3:**
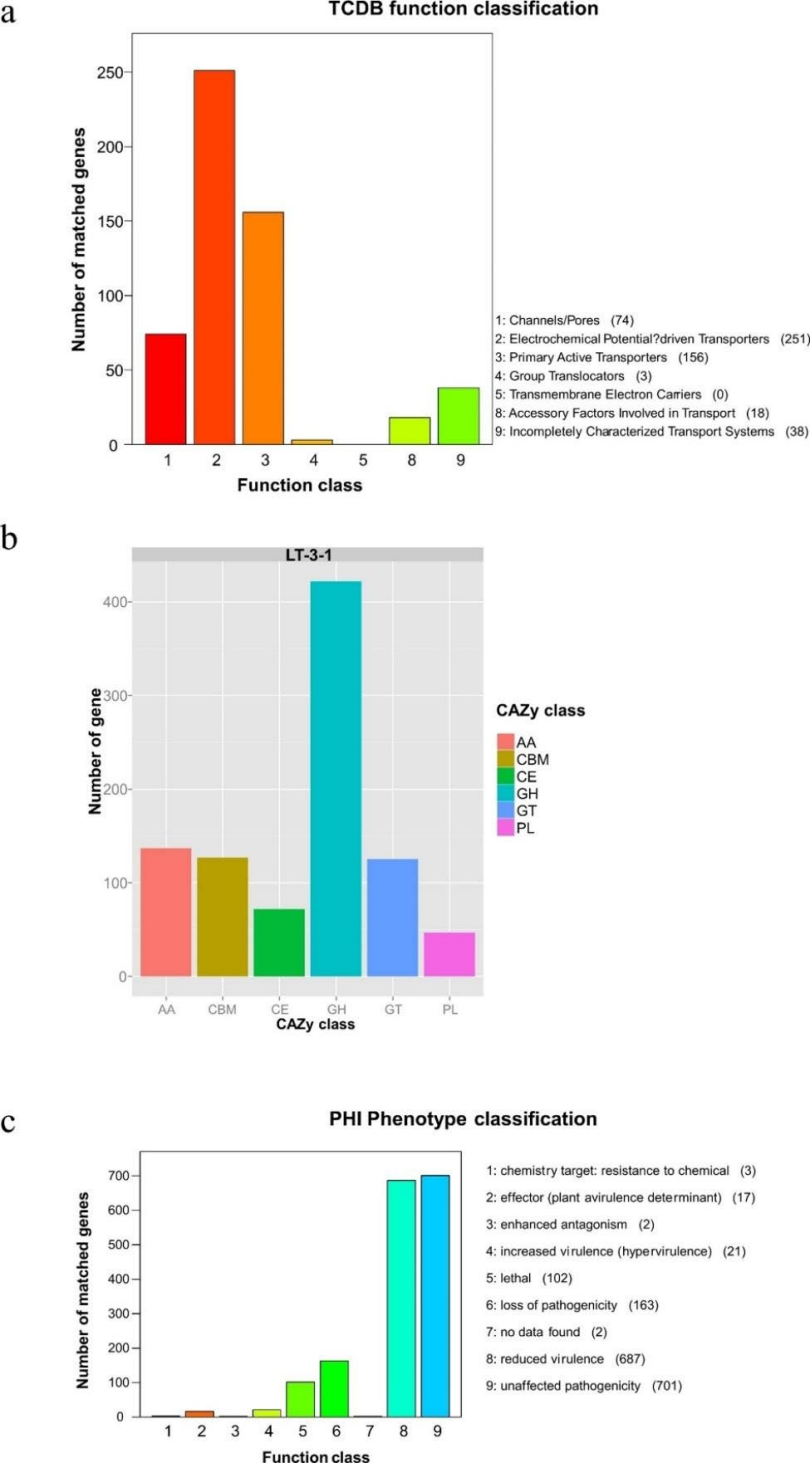
Bar graphs for the genomic clusters related to pathogenicity. a to c) The TCDB function class **(a)**, the clusters of carbohydrate-active enzymes (CAZy) functional classification of proteins **(b)**, and the PHI phenotype classification **(c)** of the *C. camelliae* genome. Abscissas and ordinate axis refer to functional classification and gene number, respectively

The Transporter Classification Database (TCDB) revealed a total of 540 genes, which include a total of 251 transporter genes driven by electrochemical potential-driven transport, primary active transporters (156 genes), channels/pores (74 genes), incompletely characterized transport systems (38 genes), accessory factors involved in transport (18 genes), and group transporters (3 genes). It is noteworthy that no transmembrane electron carrier genes were found (Fig. 3a).

#### Carbohydrate-active enzymes (CAZy)

A total of 836 genes, accounting for 5.63% of the predicted genes, were annotated as the result obtained from the CAZy database. Glycoside hydrolases (GHs) were detected to have the highest number (422 genes) of genes, followed by auxiliary activities (AAs) (137 genes), carbohydrate-binding modules (CBMs) (127 genes), glycosyltransferases (GTs) (125 genes), carbohydrate esterases (CEs) (72 genes), and polysaccharide lyases (PLs) (47 genes) (Fig. 3b). Moreover, a total of 541 genes were identified to be involved in plant cell wall hydrolases, including CEs, GHs, and PLs, the key factors for successful fungal penetration and infection for plant hosts (Table S3).

#### Secreted proteins

Several proteins (1996 secreted proteins) with a signal peptide structure were detected, including 3286 proteins with the transmembrane structure in addition to 1661 proteins with the signal peptide structure without the transmembrane structure (Table S4).

#### CytochromeP450

The data obtained from the cytochrome P450 database revealed that a total of 303 genes, accounting for 2.04% of the predicted genes, were annotated (Table S5).

#### Secondary metabolic gene clusters

A total of 71 secondary metabolic gene clusters were predicted, including 9 related to non-ribosomal peptide synthase (NRPS), 27 to polyketide synthase (PKS) (25 t1PKS and 2 t3PKS), 4 to t1PKS-NRPS, 7 to indoles, and 14 to terpenes, one to indole-t1PKS and one to terpene-NRPS (Table S6).

#### Virulence factors

According to the results obtained from the virulence factors database (DFVF), A total of 571 genes (about 3.85%) were annotated. It is worth noting that the A05828 gene of *C. camelliae* shares an identical sequence with the HIS3 gene of *Fusarium* sp., which produces mycotoxin, while the A07414 gene shares a high similarity (99.4%) with the CMK1 gene of *G. lagenarium* which causes anthracnose.

#### Pathogen-Host interactions (PHI)

The findings from the PHI database revealed that a total of 1698 (11.44%) genes were annotated (Fig. 3c, Table S7). Most of these genes were conserved in the phytopathogenic fungi; e.g., the A07414 gene of *C. camelliae* shares an identical sequence with the ChMK1 gene of *Colletotrichum higginsianum*, while the A02696 gene shares a high identity of 99.7% with the GzGPA1 gene of *Fusarium graminearum*.

### Comparative genomics analysis

#### Synteny analysis with other phytopathogenic fungi

**Fig. 4:**
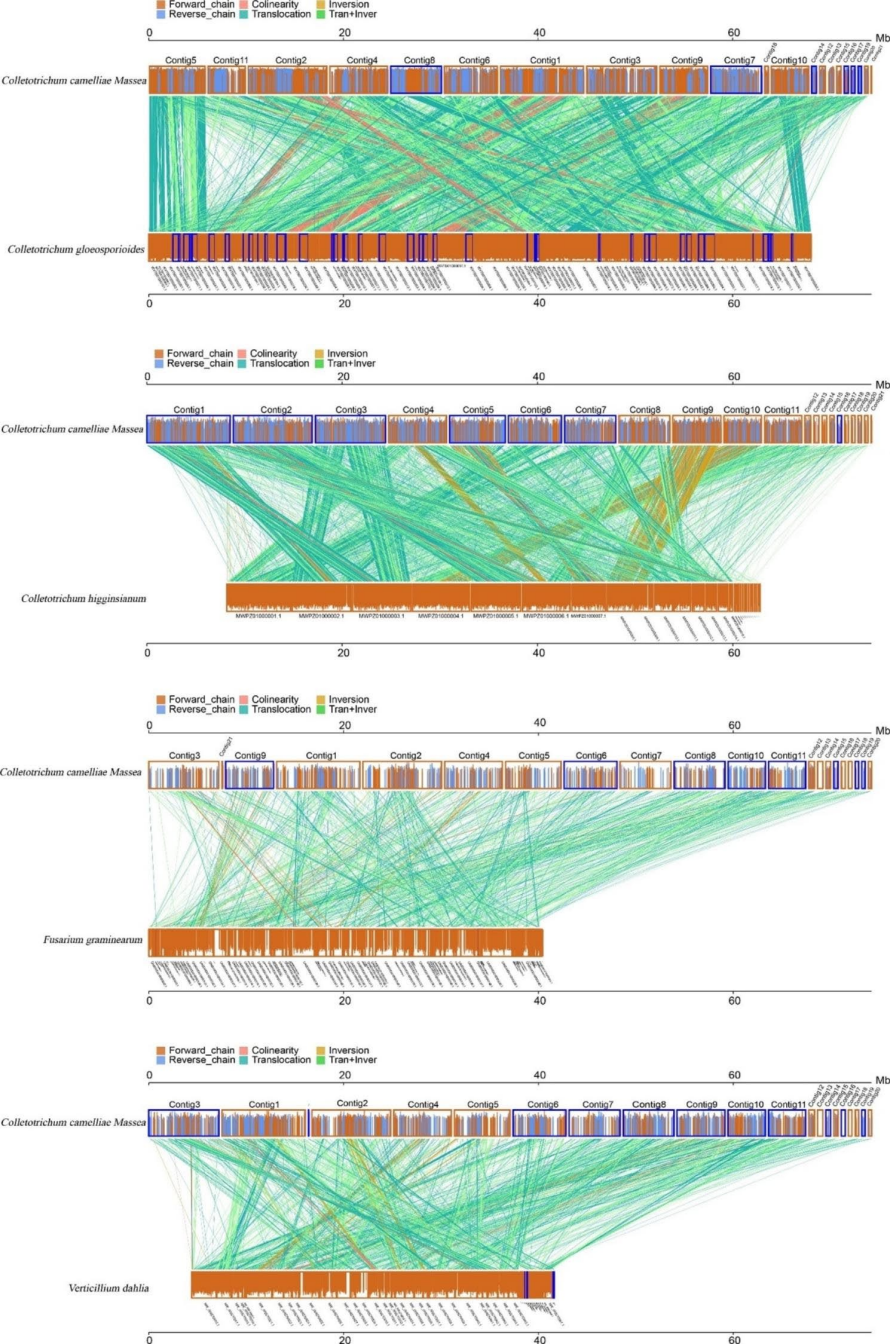
Parallel collinearity comparison between *C. camelliae, C. gloeosporioides*, *C. higginsianum*, *F. graminearum*, and *V. dahlia*

Synteny analysis was conducted between the genomic sequences of *C. camelliae* and those of closely related phytopathogenic fungi, including *C. gloeosporioides*, *C. higginsianum*, *F. graminearum*, and *Verticillium dahlia*. The findings revealed that *C. camelliae* was closely related to *C. gloeosporioides*, with 16,785 synteny blocks in size of 51.8 Mb, accounting for 76.49% of the total genomic size (Fig. 4). A total of 16,066 (accounting for 27.95% of the total genomic size), 6186 (8.82%), and 2961 (3.73%) synteny blocks were detected for the genomes of *C. camelliae* and *C. higginsianum*, *V. dahlia*, and *F. graminearum*, respectively.

#### Core- and pan-genes in comparison with other phytopathogenic fungi

**Fig. 5:**
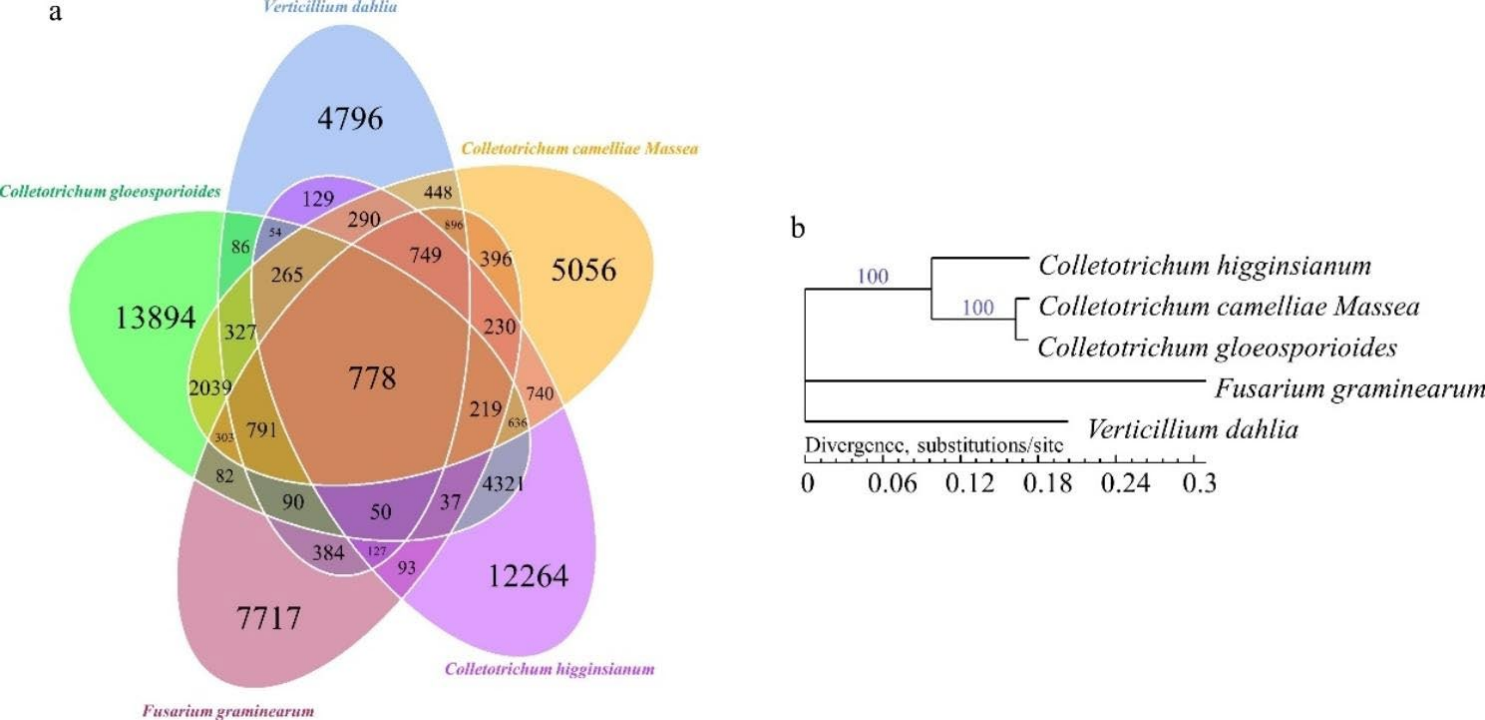
Core- and pan-genes among the genomes of *C. camelliae* and other phytopathogenetic fungi, and their phylogenetic relationship. **(a)** Venn Diagram of core- and pan-genes of *C. camelliae*, *C. gloeosporioides*, *C. higginsianum*, *F.graminearum*, and *V. dahlia*. **(b)** phylogenetic tree constructed based on the core genes of *C. camelliae*, *C. gloeosporioides*, *C. higginsianum*, *F. graminearum*, and *V. dahlia*

As compared with other fungal species, including *C. gloeosporioides*, *C. higginsianum*, *F. graminearum*, and *V. dahlia*, a total of 778 core genes conserved in the aligned fungal species were detected in *C. camelliae*. The number of core genes discovered between *C. camelliae and C. gloeosporioides, C. higginsianum, F. graminearum*, and *V. dahlia* were increased when two species were aligned, including 5358, 3907, 4362, and 4544 core genes, respectively. Contrastively, the specific genes are 8805, 10,583, 9801 and 9619 for *C. camelliae*, as compared to these fungal species, respectively (Fig. 5a).

#### Analysis of species evolution

A phylogenetic tree was constructed based on the single copy core gene, placing *C. camelliae* together with *C. gloeosporioides* and *C. higginsianum* in the same cluster. However, *C. camelliae* was more phylogenetically related to *C. gloeosporioides* than to C. *higginsianum*. (Fig. 5b). As expected, *C. camelliae* was placed in a cluster in different branches with the two fungi, *F. graminearum*, and *V. dahlia*.

## Discussion

Even though *C. camelliae* is an important pathogenetic fungus of tea plants, one of the most economically important plants worldwide, its genome has previously remained uncharacterized. The whole genome of *C. camelliae* was sequenced using the Pacbio RSII sequencing platform, one of the most advanced Third-generation sequencing platforms, with the advantages of long reads, high accuracy and sensitivity [19]. A high-quality genomic sequence was obtained as indicated by the N50 contig of *C. camelliae* with 5.6 Mb in size. However, this genomic sequence of *C. camelliae* is significantly longer than those of *C. orbiculare*, *C. gloeosporioides*, *C. higginsianum*, *C. fructicola*, *C. orbiculare*, and *C. graminicola*, which sequenced by Roche 454 and Illumina platforms with the N50 contigs in the sizes of 112.81 to 1241 kb [20–22]. It is worth noting that two genome drafts of *C. camelliae* strains LS-19 (isolated from *C. sinensis*) and CcLH18 (*C. oleifera*) have been submitted in GenBank (accession numbers of. GCA_018853505.1, Genome size 67.5 Mb, Contig N50 5.9 Mb; GCA_011947485.2, Genome size 57.84 Mb, Contig N50 unknown) without annotation. Both genomes are smaller than the current genome (67.74 Mb) of the strain LT-3-1 of *C. camelliae*. This finding might be due to incomplete assembling or strain variations. Here we provide more authentic and confident genomic information for *Colletotrichum* fungi in addition to the newly sequenced *C. lupini* genome using Pacbio sequencing platform [22].

As compared with the sequenced and annotated genomes of other *Colletotrichum* species, *C. camelliae* genomic size fits in the range of 46.76–109.7 Mb. This result reveals that the *C. camelliae* genome is smaller than those of *C. trifolii* (109.7), *C. orbiculare* (89.75), *C. sidae* (86.83), and *C. spinosum* (82.73), similar to *C. shisoi* (69.67) and *C. viniferum* (68.45), and longer than the remaining *Colletotrichum* genomes, e.g., *C. fructicola* (55.61–59.54), *C. gloeosporioides* (53.21–61.90), and *C. sublineola* (46.76). Therefore, *C. camelliae* does not reduce its genomic size although it has a narrow host range as compared with other *Colletotrichum* fungi with a broad host range, as exemplified by *C. gloeosporioides* and *C. fructicola*. Based on synteny and phylogenetic analysis, *C. camelliae* is closely related to *C. gloeosporioides*, which has a wide host range causing many anthracnose diseases of horticultural crops e.g., olive, citrus, pepper, banana, and tea plants [4, 23–25]. On tea plants, *C. gloeosporioides* causes anthracnose and leaf blight disease showing uniformed white lesions. In contrast, *C. camelliae*, with a very narrow host range, specifically infects *Camellia* plants, resulting in tea brown blight disease, which was characterized by typical symptoms of cloudy lesions with black-and-white pattern on the infected leaves. Moreover, *C. camelliae* has many unique morphological and biological traits that distinguish it from those of *C. gloeosporioides*. For *C. camelliae*, the optimum temperature is 28℃ for the growth and forming the asexual stage including large conidia [in size of (10–21) × (3–6) µm] in a large acervulus (187–290 μm in diameter) with setae, whereas the optimum growth temperature is 25℃ for *C. gloeosporioides* which produces small conidia [(3–6) × (2-2.5) µm] in a smaller acervulus (80–150 μm in diameter) without setae. Considering that *C. camelliae* has a specific host range and different morphological and biological traits as compared with *C. gloeosporioides*, the well-characterized but closely phylogenetically related species, this study provides new insights into genomic information for a better understanding of genome structure, evolution, and biological function of *Colletotrichum* fungi.

Genomic organization analysis of *C. camelliae* reveals that it is functionally separated into coding, DRs, TRs, satellite DNAs, and noncoding RNA coding regions. Among them, the putative gene number (14,854 genes, accounting for 34.01% of the whole genome) intermediates those of *Colletotrichum* fungi, ranging from 12,006 (for *C. graminicola*) to 18,324 genes (*Colletotrichum lupini*). It is worth noting that the genome of *C. camelliae* has 615 genes fewer than those of *C. gloeosporioides* and *C. fructicola*, which have a broad host range, while it has 3470 genes fewer than those of *C. lupini* with host specificity. It suggests that the fungal host range is not related to their gene numbers. We also noticed that the DRs rank at 5.89% of the whole genome of *C. camelliae*, which is markedly higher than those of *C. gloeosporioides* and *C. fructicola*. A large amount of DRs might contribute to expanding the genomic sizes of *C. camelliae*. This situation is similar to that of *C. orbiculare* which has a large genomic size (88.3 Mb) with high DRs (8.3%) and fewer genes (13,479 genes) [20]. Additionally, the genome of *C. camelliae* encodes 98 rRNAs, which is remarkably higher than those in most *Colletotrichum* species such as both *C. gloeosporioides* and *C. orbiculare* that encode 58 rRNAs.

The genome of *C. camelliae* contains a large class of genomic clusters potentially related to the fungal pathogenicity, including transporter (540 genes), CAZy (836 genes), secreted proteins (1996 genes), CYP450 (303 genes), secondary metabolic gene clusters (71 genes), and virulence factors (571 genes). A total of 1698 (11.44% of the predicted genes) genes were annotated in the PHI database, and 541 genes were related to plant cell wall hydrolase, which is remarkably higher than those of both *C. gloeosporioides* (327 genes) and *C. orbiculare* (346 genes), as well as those of other hemibiotrophic and necrotrophic fungi, including *Fusarium* spp., *Magnaporthe oryzae*, *Botrytis cinerea*, and *Sclerotinia sclerotiorum* [20, 21, 26–28]. Consistent with this, a large number of genes (422 genes) belonging to the glycoside hydrolase (GH) family, including GH3 (29), GH16 (27) and GH18 (21) in the top three, were enriched in the genome of *C. camelliae*, and these gene clusters are concluded to be important pathogenetic factors of *C. camelliae*, which was identified to be responsible for the fungal infection and pathogenicity. Similarly, GHs 6, 7, and 76 families play an important role in the virulence of *M. oryzae* [29, 30]. Since the plant cell wall hydrolases of phytopathogenic fungi behave as the pathogenetic key factors to break down the cell walls and establish infection and saprophytic growth, the expansion of cell wall degrading enzymes in the *C. camelliae* genome most likely reflects an important pathogenic factor to infect tea plants.

With knockout-backup approaches, other pathogenic factors have been identified in *C. gloeosporioides*, including cutinase, laccase, bZIP transcription factor, calcium-translocating P-type ATPase and other pathogenicity genes [31–34]. All these genes are also detected in *C. camellia* and have high identities with those of *C. gloeosporioides*, suggesting that they are most likely to have the same functions. Additionally, since the ChMK1 gene has been identified to be responsible for the fungal pathogenicity causing anthracnose leaf spot as well as playing an important role in growth, cell wall integrity, and colony melanization [35], and the A07414 gene (mitogen-activated protein kinase) in *C. camelliae* is identical with the ChMK1 gene of *C. higginsianum*, which is most likely to be one of the key pathogenetic factors of tea brown blight causing the cloudy symptoms. In addition, a large number of genes belong to the major facilitator superfamily (MFS), another class of pathogenic factors in pathogenic fungi [36] were detected in *C. camelliae*, which might contribute to the delivery of some virulence factors or compounds, sugars, amino acids, vitamins, drugs and other small molecules to plants and environment supporting the process of the fungal infection. Moreover, *C. camelliae* appears to have an even greater capacity (with 71 gene clusters) for secondary metabolite production, similar to those of *C. gloeosporioides* (76 gene clusters), fewer than those of *C. higginsianum* (84 gene clusters), higher than those of both *C. orbiculare* (54 gene clusters) and *C. graminicola* (53 gene clusters), and much higher than those identified from other fungal genomes such as *M. oryzae* (32 gene clusters) and *F. graminearum* (37 gene clusters). In addition, *C. camelliae* encodes a large number of cytochrome P450 proteins, accounting for 2.04% of the total genes, remarkably higher than those in *C. gloeosporioides* (1.06%) and *C. orbiculare* (0.74%), respectively. These proteins are related to both primary and secondary metabolisms, and the production of mycotoxins, and detoxification [37]. Finally, it is worth noting that the synteny (accounting for 76.49% of the total genomic size) between *C. camelliae* and *C. gloeosporioides* revealed that both species were placed together in the same cluster based on the phylogenetic analysis. This finding reveals that the synteny between *C. camelliae* and *C. gloeosporioides* is remarkably higher than that between *C. graminicola* and *C. higginsianum* (35%) and that between *C. orbiculare* and *C. fructicola* (40%) (Gan et al. 2013; O’Connell et al. 2012). These results suggest that *C. camelliae* most likely evolved from *C. gloeosporioides* through the expansion of some specific pathogenicity-related gene clusters, which might be related to its specific host range and pathogenicity differing from other *Colletotrichum* fungi.

## Conclusion

A high-quality genome of *C. camelliae* was sequenced by the Pacbio RSII platform, and its pathogenicity-related gene clusters were annotated. A large class of plant cell wall hydrolases was detected, indicating the number remarkably higher than those of *C. gloeosporioides* and *C. orbiculare*, as well other hemibiotrophic and necrotrophic fungi. The expansion of cell wall degrading enzymes most likely reflects an important adaptive trait to infect tea plants. Moreover, other pathogenic factors including cutinase, laccase, bZIP transcription factor, calcium-translocating P-type ATPase, and other pathogenicity genes are also detected in *C. camelliae*, suggesting that they have high identities with those of *C. gloeosporioides*, sharing the same functions. Both synteny relationship and phylogenetic analysis revealed that *C. camelliae* is closely related to *C. gloeosporioides*, while it contains a large class of diverse genes or other functional clusters separating *C. camelliae* from other *Colletotrichum* fungi.

Collectively, *C. camelliae* has a specific host range and unique morphological and biological traits compared to other *Colletotrichum* fungi. Thus, characterization of the fungal genome will provide us with substantial data and a theoretical basis for studies on the transcriptome, proteome, and metabolome as well as enrich the database of *Colletotrichum*.

## Methods

### Fungal materials and cultural conditions

A phytopathogenic strain LT-3-1 of *C. camelliae* inducing brown leaf blight was isolated from tea plants cultivated in Hubei Province, China [38]. Fungal mycelia were cultured on potato dextrose agar (PDA) medium at 25℃ for 6 days, collected, frozen in liquid nitrogen, and then stored at -80℃ until use for further experiments.

### Genome sequencing and assembly

Genomic DNA was extracted using the SDS-based DNA extraction method as previously described [39]. Extracted DNAs were fractioned by 1% agarose gel electrophoresis and detected by UV transillumination after staining with ethidium bromide (0.1 mg/mL), quantified by Qubit, and then sequenced using the Single Molecule Real-Time (SMRT) technology [40] by Beijing Novogene Bioinformatics Technology Co., Ltd. The low quality reads were filtered and the filtered reads were assembled to generate one contig without gaps by the SMRT Link v5.0.1.

### Genome component prediction

The genomic information of *C. camelliae* was predicted with AUGUSTUS v2.7 [41, 42] and the homologous GeneWise tool as referring to the homologous protein sequence of *C. gloeosporioides* (accession No. QFRH00000000) to predict the coding genes, repetitive sequences, and non-coding RNAs. The coding genes were generated using PASA after the predicted data generated with both tools were integrated with EVM. The interspersed repetitive sequences and the tandem repeats were predicted using the RepeatMasker (http://www.repeatmasker.org/) [43] and the Tandem repeats finder (TRF) [44], respectively. Moreover, the transfer RNAs (tRNAs), ribosomal RNAs (rRNA), small RNA (sRNAs), small nuclear RNAs (snRNAs), and microRNAs (miRNAs) were predicted by tRNAscan-SE [45], rRNAmmer [46], and BLAST against the Rfam database [47], respectively.

### Gene function annotation

Gene function was annotated by BLAST search (E-value less than 1e^− 5^, minimal alignment length percentage larger than 40%) with the sequences reported in seven databases, including Gene Ontology (GO), Kyoto Encyclopedia of Genes and Genomes (KEGG)[48], Clusters of Orthologous Groups (KOG), Non-Redundant Protein Database databases (NR), Transporter Classification Database (TCDB), cytochromeP450, Pfam and Swiss-Prot. Moreover, the secretory proteins were predicted based on the alignment with SignalP database [49], while the secondary metabolism gene clusters by antiSMASH [50]. Pathogenicity- and virulence-related genes or factors were analyzed based on Pathogen Host Interactions (PHI) and fungal virulence factors (DFVF) databases, respectively. However, Carbohydrate-Active enzymes were analyzed using Carbohydrate-Active enZYmes Database (CAZy).

### Comparative genomics analysis

The genomes of *C. gloeosporioides* (accession No. WVTB00000000), *Colletotrichum higginsianum* (accession No. MWPZ00000000), *Fusarium graminearum* (accession No. CABDWO000000000), and *Verticillium dahliae* (accession No. ABJE00000000), were selected for comparison with the genomic data of *C. camelliae*. Genomic alignment was performed using the MUMmer and LASTZ tools [51, 52], and then the genomic synteny was analyzed. Both core and specific genes were analyzed by the CD-HIT rapid clustering of similar proteins software with a threshold of 50% pairwise identity and 0.7 length difference cutoff in amino acid[53]. The phylogenetic tree was constructed by the PhyML and the setting of bootstraps was 1,000 with the orthologous genes as previously described [54].

## Electronic supplementary material

Below is the link to the electronic supplementary material.

Supplementary Material 1

Supplementary Material 2

## Data Availability

The *Colletotrichum camelliae* genomic data has been deposited at DDBJ/ENA/GenBank repository under the accession JAPPVZ000000000, under the linkage (https://www.ncbi.nlm.nih.gov/nuccore/JAPPVZ000000000). The version described in this paper is version JAPPVZ010000000. The reported assemblies are associated with NCBI BioProject: PRJNA907149 and BioSample: SAMN31952954 within GenBank.
