# Supplementary figures and images for: Genomic analysis of Colletotrichum camelliae responsible for tea brown blight disease
Source: BMC Genomics. 2023 Sep 6;24:528. doi: 10.1186/s12864-023-09598-6 (PMC10483846; doi:10.1186/s12864-023-09598-6)

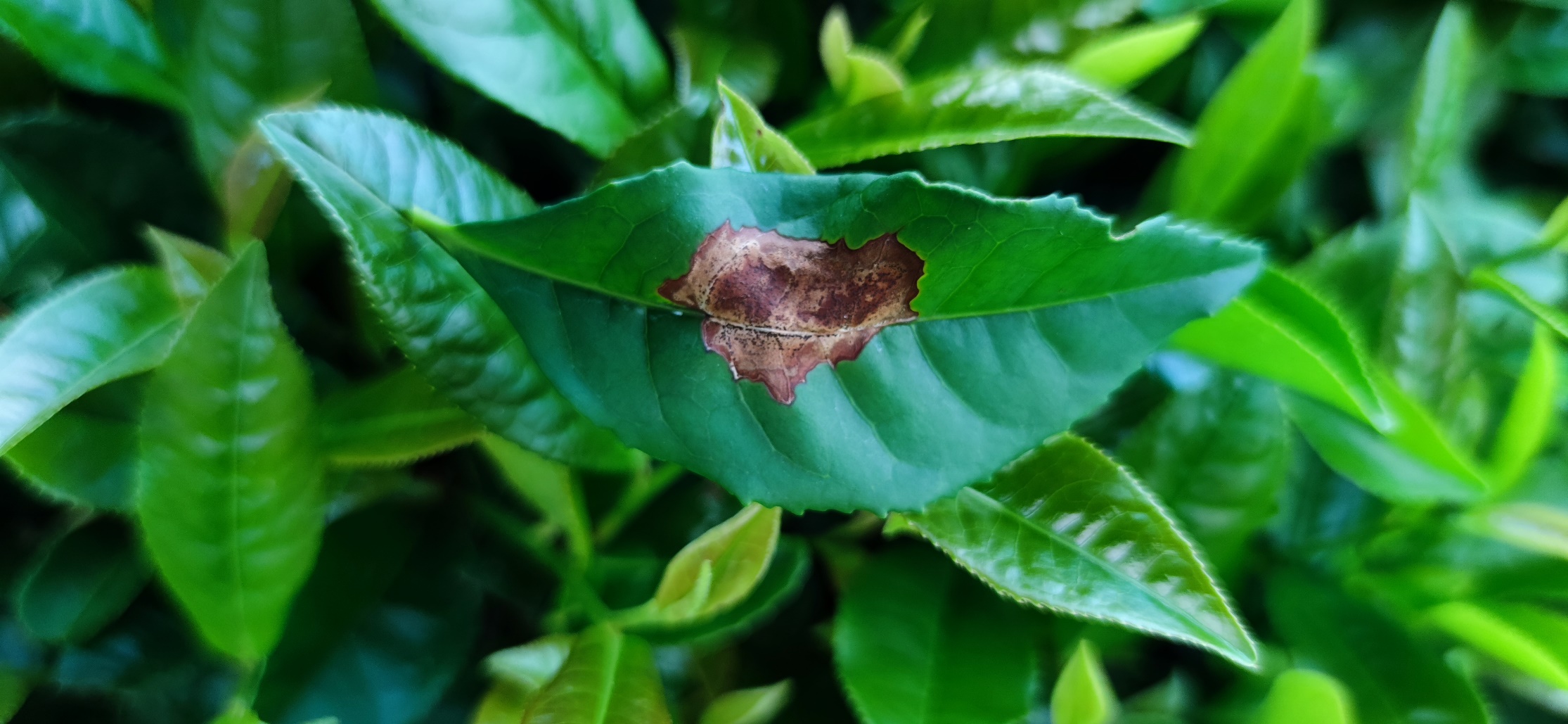


Figure S1 Typical brown blight symptoms on a tea leaf induced by *Colletotrichum camelliae*.

Supplement: Supplementary file 2 — Supplementary Material 2 [file 12864_2023_9598_MOESM2_ESM.docx]
